# Chronic unpredictable mild stress induces anxiety-like behavior in female C57BL/6N mice, accompanied by alterations in inflammation and the kynurenine pathway of tryptophan metabolism

**DOI:** 10.3389/fnins.2025.1556744

**Published:** 2025-02-26

**Authors:** Yanqin Luo, Ning Jiang, Yiwen Zhang, Yongzhi Zhao, Fang Chen, Xueyan Li, Meng Qiang, Guirong Zeng, Qinghu He, Xinmin Liu, Chunhui Shan

**Affiliations:** ^1^Engineering Research Center of Storage and Processing of Xinjiang Characteristic Fruits and Vegetables, Ministry of Education, School of Food Science, Shihezi University, Shihezi, Xinjiang, China; ^2^Sino-Pakistan Center on Traditional Chinese Medicine, Hunan University of Medicine, Huaihua, China; ^3^Research Center for Pharmacology and Toxicology, Institute of Medicinal Plant Development (IMPLAD), Chinese Academy of Medical Sciences and Peking Union Medical College, Beijing, China; ^4^Institute of Drug Discovery Technology, Ningbo University, Ningbo, China

**Keywords:** chronic unpredictable mild stress (CUMS), anxiety, inflammation cytokines, kynurenine pathway, female, mice

## Abstract

Chronic stress can impact brain function through various mechanisms, contributing to the development of anxiety disorders. Chronic unpredictable mild stress (CUMS) is a well-established model for studying the effects of chronic stress. This study assessed the impacts of different durations of CUMS on anxiety-like behavior, inflammation, and tryptophan metabolism in female C57BL/6N mice. The results revealed significant behavioral changes after 2–4 weeks of CUMS. Specifically, the open arms ratio and open arms time ratio in the elevated plus maze (EPM) decreased, the latency to feed in the novelty-suppressed feeding test (NSFT) was prolonged, and the number of transitions in the light/dark box (LDB) was decreased. After 1 week of CUMS, the levels of some pro-inflammatory cytokines (such as IL-1β and iNOS) and anti-inflammatory cytokines (including IL-10) began to rise. After 2 weeks of CUMS, most pro-inflammatory cytokines (IL-1β, IL-6, CD86, iNOS) and the anti-inflammatory cytokines TGF-β and CD11b showed an increase, while some anti-inflammatory cytokines (Arg-1, IL-10) began to decrease. After 3 weeks of stress, the pro-inflammatory cytokine TNF-α also significantly increased, while the anti-inflammatory cytokine TGF-β began to decline. By 4 weeks of CUMS, the anti-inflammatory cytokine CD11b also started to decrease. Regarding tryptophan metabolism, after 3–4 weeks of CUMS, serotonin (5-HT) levels in the hippocampus of the mice began to decrease. Additionally, the kynurenine pathway in tryptophan metabolism shifted more towards the KYN-QA branch, resulting in the reduction in the neuroprotective substance kynurenic acid (KYNA), while neurotoxic substances such as 3-hydroxykynurenine (3-HK) and quinolinic acid (QA) accumulated. In summary, female C57BL/6N mice exhibit anxiety-like behavior after 2 weeks of CUMS, accompanied by inflammatory responses. After 3–4 weeks of CUMS, anxiety-like behavior persists, with exacerbated inflammatory responses and disturbances in tryptophan metabolism.

## 1 Introduction

Stress refers to the non-specific response generated by the body when stimulated by strong internal and external factors. It is an adaptive mechanism that the body employs to maintain internal environmental homeostasis and support normal physiological functioning (Szabo et al., 2012). Prolonged stress can have profound effects on both physiological and psychological health, potentially leading to the development of anxiety disorders (Biltz et al., 2022). Anxiety disorders are the most common mental health disorders in the world, second only to depression. These disorders are characterized by excessive fear or worry, either in specific situations or in daily life, and they significantly impact human health, contributing to a substantial medical burden (Penninx et al., 2021). It is expected that by 2030, it would cause a great number of economic losses (Zhang et al., 2024). In recent years, the incidence of anxiety disorders has been rising annually. Current estimates suggest that approximately 3.1% of the global population suffers from anxiety disorders, with a lifetime prevalence reaching up to 5.7%. Currently, commonly used medications for treating anxiety disorders include benzodiazepines, selective serotonin reuptake inhibitors (SSRIs), and tricyclic antidepressants (TCAs). However, long-term use of these medications may lead to patients developing tolerance, dependence, and risk of abuse, and withdrawal symptoms may occur upon discontinuation (Slee et al., 2019). Therefore, in-depth research into the pathogenesis of anxiety disorders and the development of new medications is particularly urgent to explore more effective treatment methods, improve treatment outcomes, and reduce side effects.

Research shows that anxiety disorders exhibit notable gender differences, with the prevalence in females being 1.66 times that of males (Santomauro et al., 2021; Javaid et al., 2023). Among anxiety disorders, female patients more commonly complain of physical symptoms such as fatigue, muscle tension, and cardiopulmonary and gastrointestinal discomfort (Altemus et al., 2014). Despite the higher prevalence and severity of anxiety disorders in females, most current research on the neurobiological mechanisms underlying anxiety and depression has been conducted primarily in male animal models. This has resulted in a limited understanding of the mechanisms in females and a lack of tailored treatment assessments for female patients. Therefore, establishing female-specific animal models of anxiety disorders is essential for investigating gender-related differences in circuits and behavioral mechanisms, as well as for developing targeted treatments that address the unique needs of female patients.

The pathogenesis of anxiety disorders is complex, and it is generally believed that the occurrence of anxiety disorders is related to an imbalance of various neurotransmitters. Serotonin (5-HT), norepinephrine (NE), dopamine (DA), glutamate (Glu) and gamma-aminobutyric acid (GABA) play key roles in regulating mood and stress responses in the brain (Mittal et al., 2017). When the balance of these neurotransmitters is disrupted, the normal information transmission function in the brain is affected, leading to emotional dysregulation and triggering symptoms of anxiety (Zhao et al., 2023). In addition to neurotransmitter imbalances, neuroinflammation is also considered an important risk factor for the development of anxiety disorders (McKim et al., 2018). Inflammatory responses can affect brain function, including the synthesis, release, and metabolism of neurotransmitters, thereby influencing emotional regulation. Chronic stress, sustained over time, can increase inflammation levels in the brain and periphery, activating the immune system and increasing the release of cytokines (Glaser and Kiecolt-Glaser, 2005; Biltz et al., 2022). Pro-inflammatory cytokines can activate the kynurenine pathway in tryptophan metabolism, resulting in the accumulation of neurotoxic substances such as 3-HK and QA. The accumulation of these neurotoxic substances further exacerbates the inflammatory response, negatively impacting brain function, and thereby triggering or worsening anxiety disorders (Foster et al., 1985, 1986; Dehhaghi et al., 2019; Mithaiwala et al., 2021).

CUMS is a widely used model to simulate chronic stress in humans over an extended period, often used to study depression. Additionally, CUMS can also induce anxiety-related behaviors and physiological changes, making it widely used in anxiety disorder research. This study selected female C57BL/6N mice to assess the cycle of anxiety-like behaviors induced by CUMS stress and employed quantitative fluorescent PCR to detect the expression levels of inflammatory factors in the hippocampus of the mice. Ultra-high-performance liquid chromatography–tandem mass spectrometry (UPLC–MS/MS) was used to detect changes in tryptophan metabolites in the hippocampus of the mice. The goal was to establish a stable and reliable CUMS-induced anxiety-like behavior model in female C57BL/6N mice and determine the relationship between inflammation, tryptophan metabolism, and anxiety in female mice.

## 2 Materials and methods

### 2.1 Chemical reagent

TRIzon (total RNA extraction) reagents were purchased from Beijing Kangwei Century Biotechnology Co., Ltd., PerfectStart Green qPCR SuperMix and TransScript^®^ All-in-One First-Strand cDNA Synthesis SuperMix for qPCR (One-Step gDNA Removal) were purchased from Beijing Quanshijin Technology Co., Ltd., TRP, 5-HT, KYN, KYNA, 3-HK, QA, DA, NE, Glu, GABA were purchased from Sigma.

### 2.2 Animals

Eighty (80) female C57BL/6N mice (6 weeks old) were purchased from Beijing Vital River Laboratory Animal Technology Co., Ltd. The animals were housed in an environment with a temperature of 23 ± 2°C, humidity of 50 ± 5%, and a 12-h light/dark cycle. All mice were free access to food and water and were acclimatized to these conditions for 5 days. All procedures were performed under the National Institutes of Health Guide for the Care and Use of Laboratory Animals. All the animal experiments were approved by the Animal Ethics Committee of the Institute of Medicinal Plant Development, Chinese Academy of Medical Sciences (approval no. 20161028).

### 2.3 Experimental design

**Figure 1:**
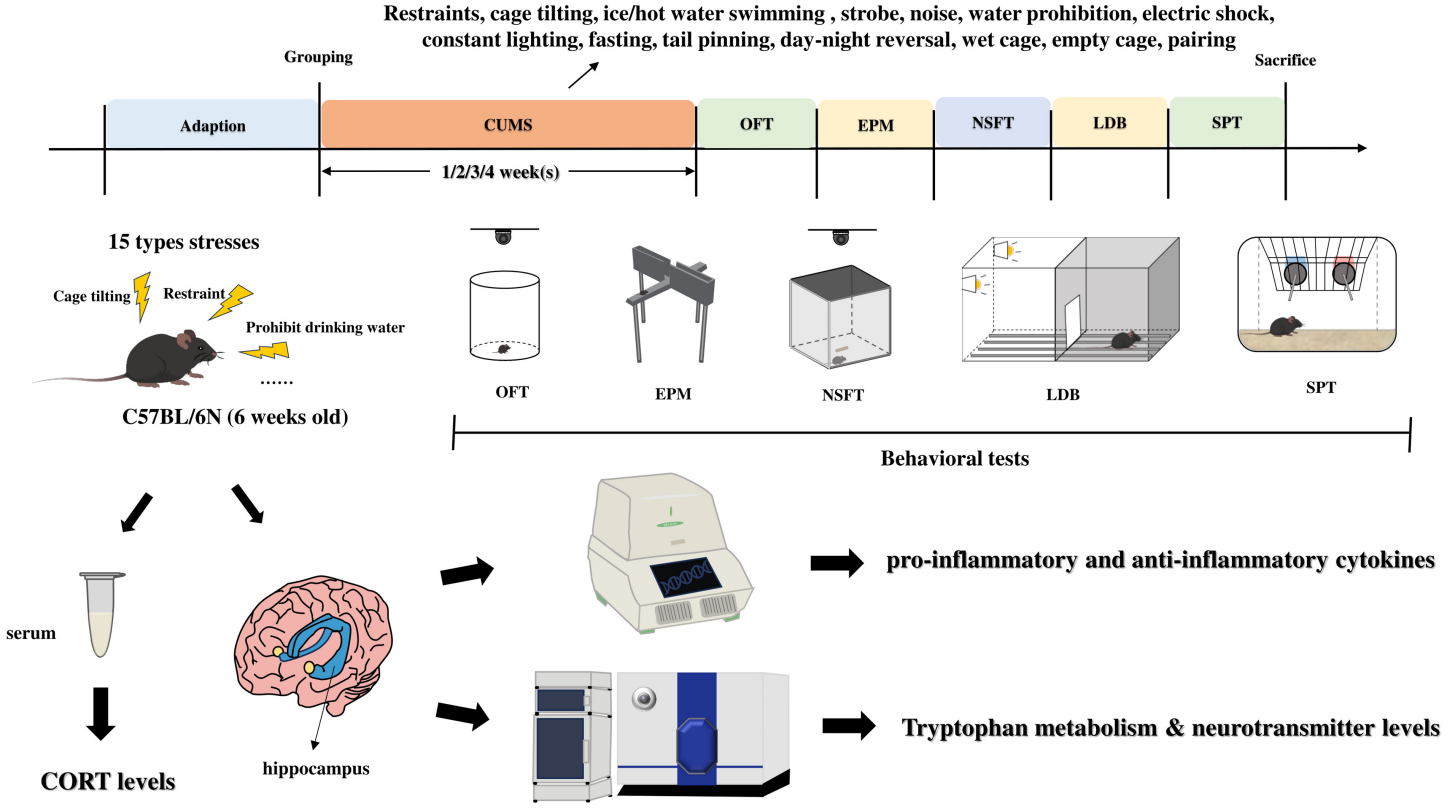
Experimental procedure. CUMS, chronic unpredictable mild stress; OFT, open field test; EMP, elevated plus maze; NSFT, novelty-suppressed feeding test; LDB, light/dark box; SPT, sucrose preference test.

Eighty female C57BL/6N mice (6 weeks old) were selected for the experiment and randomly divided into 8 groups based on body weight, namely: one-week control group, one-week model group, two-week control group, two-week model group, three-week control group, three-week model group, four-week control group, and four-week model group. The control groups housed 3–4 mice per cage, while the model groups were housed individually. The model groups were exposed to the corresponding stimulus factors daily, and behavioral tests were conducted after the modeling was completed. After the behavioral experiments, serum samples and hippocampal tissues were collected for subsequent analysis (Figure 1).

### 2.4 Chronic unpredictable mild stress schedule

The stimulating factors of chronic unpredictable mild stress and daily arrangements are shown in Table 1.

**Table 1:** Chronic unpredictable mild stress schedule.

Time	The stimulating factors
Day 1	Restraints 6 h, cage tilting 12 h
Day 2	Ice water swimming 5 min, strobe light 12 h, noise 30 min, water restriction 12 h
Day 3	Electric shock 5 min, fasting 12 h, continuous lighting
Day 4	Clip on tail 3 min, cage tilting 12 h, pairing 12 h
Day 5	Fasting 12 h, wet cage 12 h, day and night reversed.
Day 6	Empty cage 12 h, water restriction 12 h
Day 7	Hot water swimming 5 min, strobe light 12 h, noise 30 min

### 2.5 Behavioral test

#### 2.5.1 Open field test

The open field test utilizes the exploratory activities of animals in response to a new open environment, and the experimental method is consistent with previous studies (Jiang et al., 2022). One hour prior, the animals are transferred to the testing room, and during the test, the animals are placed at the center of a cylinder, after adapting for 3 min in the test box, then allowing them to freely explore for 5 min. Using image recognition tracking algorithms, the distance traveled and time spent moving are recorded and analyzed in real-time to assess the motor function of the mice.

#### 2.5.2 Elevated plus maze

The elevated plus maze was tested by following previous work (Ye et al., 2024). The apparatus consists of 2 open arms (50 cm × 10 cm), 2 closed arms (50 cm × 10 cm), and a central area (10 cm × 10 cm), elevated about 50 cm off the ground. Animals are acclimated to the experimental room for 1 h before testing, or the test is started immediately after the completion of the open field test. During the test, the mice face the open arms and have their backs to the closed arms, placed in the central area. A video tracking system is used to record and analyze the time and percentage of entries into the open arms, with a testing duration of 5 min. A shorter time and percentage of entries into the open arms indicate a higher level of anxiety. After each test, the apparatus is cleaned with 75% alcohol to remove any residual feces and urine from the mice.

#### 2.5.3 Novelty-suppressed feeding test

The novelty-suppressed feeding test was conducted as previously described (Laaziz et al., 2022). It assesses the anxiety-like behavior of mice through the psychological conflict between the desire to eat generated after fasting and the fear of a novel environment. After fasting the mice (without water) for 24 h, Animals are acclimated to the experimental room for 1 h before testing. Identical food was placed in the center of the testing box (50 cm × 36 cm × 20 cm), facing away from the box. The time it took for the mice to first pick up and gnaw at the food was recorded as the latency to feed. The latency to feed was recorded within 5 min, and if the mice did not gnaw at the food within 5 min, it would be recorded as 5 min.

#### 2.5.4 Light/dark box

The light/dark box experiment made some modifications to previous experimental methods (Chen et al., 2018). It utilizes the fact that rodents prefer to explore novel environments but detest the brightness of the light room, which forces them to retreat, thus creating a state of conflict. The dimensions of the light box and dark box are 20 cm × 15 cm × 40 cm (length × width × height), with the illumination in the light room set at 300–1,000 lx. The fewer transitions, the higher the anxiety-like level of the mice. One hour before the experiment, the animals are transferred to the experimental room and placed in the center of the light room, facing the dark room, and the number of transitions is observed within 10 min.

#### 2.5.5 Sucrose preference test

The sucrose preference test is conducted according to previous descriptions (Jiang et al., 2019). The experiment is divided into a sucrose water adaptation period and a testing period, with the first 2 days as the adaptation period and the third day as the testing period. During the sucrose water adaptation period, mice are given one bottle of 1% sucrose water and one bottle of pure water, with the positions of the water bottles swapped every 12 h to eliminate position preference. After the adaptation period, testing is conducted; the animals are water-deprived for 8 h, then given pure water and 1% sucrose water for free drinking for 16 h, with the positions of the water bottles swapped midway, measuring the amount of pure water and sucrose water consumed by the animals, and calculating the sucrose preference index.

### 2.6 Sample collection

**Figure 2:**
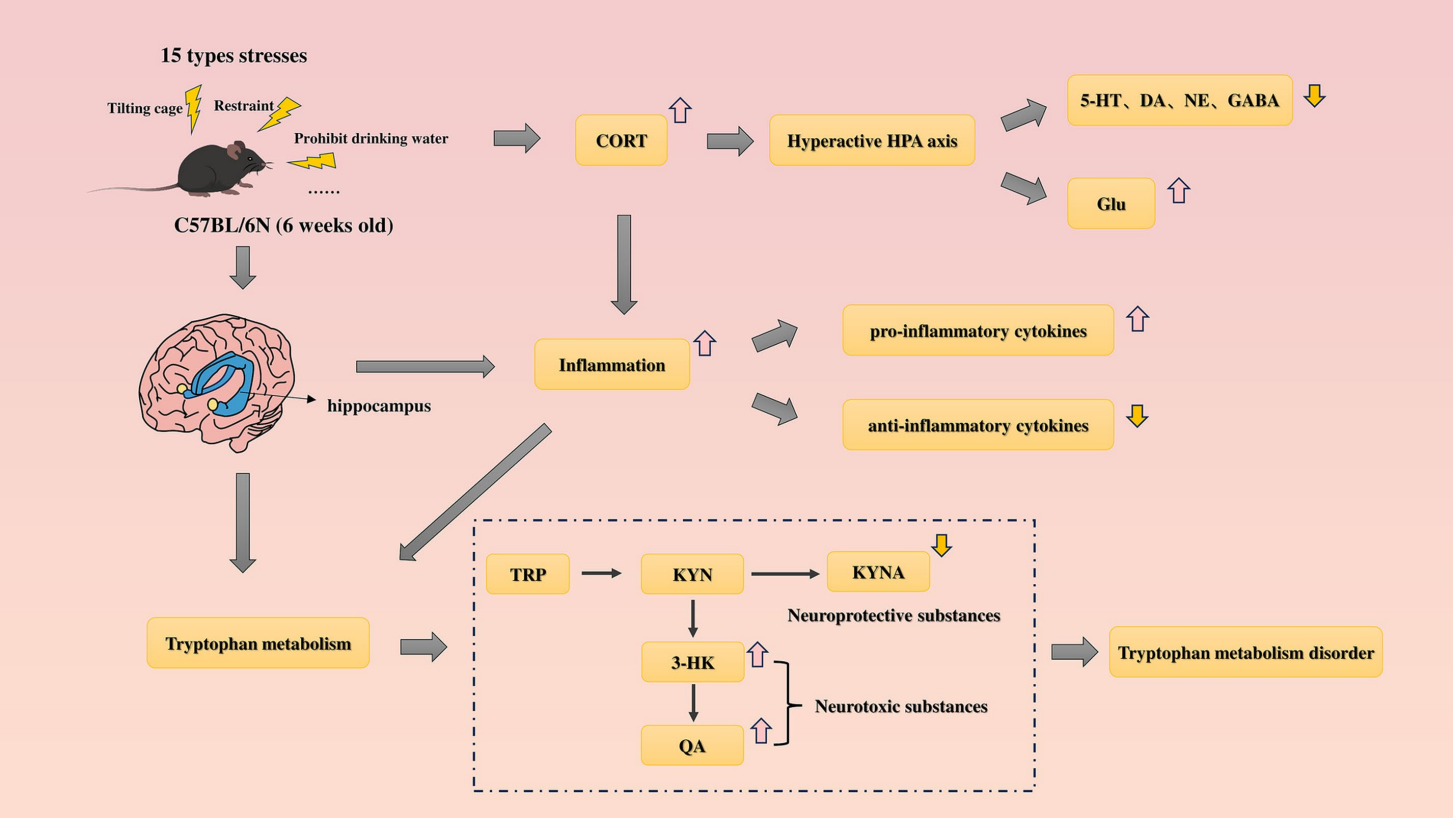
Hypothetical mechanism analysis diagram of this study.

Immediately euthanize the animals after the SPT is completed. Mice were anesthetized with pentobarbital sodium, and after removal of the eyeballs, blood was collected. The hippocampal tissue was separated after decapitation. The blood was allowed to stand overnight in a refrigerator at 4°C, and the serum was collected after centrifugation at 3,000 rpm for 15 min at 4°C in the next day and stored at −80°C for later use; the separated hippocampal tissue was placed in cryopreservation tubes, immersed in liquid nitrogen, and then transferred to a −80°C refrigerator for future experiments (Figure 2).

### 2.7 Determination of CORT levels in serum

Mouse serum was removed from the −80°C refrigerator and placed on ice to melt, after melting, it was mixed using a vortex. Remove the ELISA kit (Nanjing Jiancheng, H205-1-2) from the refrigerator in advance and place it at room temperature for half an hour to equilibrate, operate according to the instructions, and finally use an enzyme meter to detect the absorbance value of the samples at 450 nm wavelength to calculate the CORT content.

### 2.8 Determination of the mRNA relative expression levels of inflammation cytokines in the hippocampus

#### 2.8.1 RNA extraction

Mouse hippocampal tissue was weighed and placed on ice. Add 1 mL of TRIzon for every 30–50 mg of tissue, grind and mash thoroughly, vortex and mix well, and let stand at room temperature for 5 min. Add chloroform (0.2 mL of chloroform for every 1 mL of TRIzon), vortex to mix evenly, and let it sit at room temperature for 2–3 min. Centrifuge at 4°C, 12,000 rpm for 15 min, carefully aspirate the upper liquid and add an equal volume of isopropanol, vortex to mix thoroughly and let it sit at room temperature for 10 min. Centrifuge at 4°C, 12,000 rpm for 10 min, discard the liquid in the tube, and then wash the RNA three times with 75% ethanol, centrifuging at 4°C, 12,000 rpm for 3 min after each wash. After the final wash, air dry the liquid in the tube at room temperature, add RNase-Free ddH_2_O to dissolve the RNA, check the RNA concentration, and use RNase-Free ddH_2_O to adjust the RNA concentration to be consistent.

#### 2.8.2 Reverse transcription

Add reagents according to the instructions of the reverse transcription kit to make a 20 μL system. Incubate at 42°C for 15 min, then at 85°C for 5 s to inactivate it, resulting in reverse transcribed cDNA. Add RNase-Free ddH_2_O to dilute 5 times and store at −80°C for future use.

#### 2.8.3 Amplification

After adding the reverse transcribed cDNA to the amplification system, a quantitative fluorescence PCR experiment was conducted. The system was pipetted into a DNase/RNase-free 96-well plate, sealed with a film, mixed by shaking and briefly centrifuged. The qPCR reaction was performed in the CFX96, and after the reaction was completed, the expression level of the target gene was analyzed using the 2^−∆∆CT^ method, with GAPDH as the housekeeping gene (see Table 2).

**Table 2:** Primer sequences in qPCR.

Primer name	Primer sequence (5′ → 3′)
IL-1β-F	CTCGCAGCAGCACATCAACAAG
IL-1β-R	CCACGGGAAAGACACAGGTAGC
IL-6-F	TTCTTGGGACTGATGCTGGTGAC
IL-6-R	GTGGTATCCTCTGTGAAGTCTCCTC
TNF-α-F	TGTCTACTCCCAGGTTCTCTT
TNF-α-R	GCAGAGAGGAGGTTGACTTTC
iNOS-F	GGAATCTTGGAGCGAGTTGT
iNOS-R	CCTCTTGTCTTTGACCCAGTAG
CD86-F	TATCTGCCGTGCCCATTTAC
CD86-R	GTGCTCGTACAGAACCAACT
Arg1-F	TCATGGAAGTGAACCCAACTC
Arg1-R	CGAAGCAAGCCAAGGTTAAAG
CD206-F	GGCGAGCATCAAGAGTAAAGA
CD206-R	CATAGGTCAGTCCCAACCAAA
IL-10-F	CTCGCAGCAGCACATCAACAAG
IL-10-R	CCACGGGAAAGACACAGGTAGC
TGF-β-F	GCTTCTCCCAAGTGTGTCAT
TGF-β-R	GACTGCTGGTGGTGTATTCTT
CD11b-F	GCTTCAGAGATGACCAGTAAGG
CD11b-R	ACAGGGATCCAGAAGACTACA
GAPDH-F	AACAGCAACTCCCACTCTTC
GAPDH-R	CCTGTTGCTGTAGCCGTATT

### 2.9 Determination of tryptophan metabolites and neurotransmitter levels

#### 2.9.1 Processing of hippocampal samples

After weighing the hippocampus tissue, it was placed in a 1.5 mL EP tube, and 80 μL of ultrapure water was added. The mixture was homogenized using an ultrasonic disruptor in an ice-water bath. 50 μL of the homogenate was taken, and 20 μL of standard IS solution (500 ng/mL) was added, mixed by vortexing, followed by the addition of 10 μL of trifluoroacetic acid, and immediately mixed by vortexing. The mixture was centrifuged at 4°C and 20,000×*g* for 15 min. 40 μL of the supernatant was taken and stored in a refrigerator at 4°C for use the next day.

#### 2.9.2 UPLC–MS/MS method

The experimental program is guided by the methods established in the early stages (Wang et al., 2019). Before analyzing the samples, the instrument’s accuracy, repeatability, and the linear range of the standard curve were evaluated to meet the requirements for UPLC-MS/MS analysis. Restek Ultra Aqueous C18 column (100 mm × 2.1 mm, 3 μm) was used, equipped with a QTRAP 5500 mass spectrometer (AB SCIEX, Foster City, California) and a Shimadzu Prominence LC system (Kyoto, Japan) for LC-MS/MS analysis. Mobile phase A was acetonitrile, and B was a 0.1% formic acid aqueous solution. Mass spectrometry analysis was performed using electrospray ionization, with the electrospray voltage set at 4500 V, ion source temperature at 500°C, and curtain gas, auxiliary gas, and nebulizer gas maintained at 30 psi, 40 psi, and 60 psi, respectively.

### 2.10 Statistics and analysis

All results are expressed as mean ± standard error of the mean (SEM). Data analysis was performed using SPSS Statistics 26, and GraphPad Prism 8.0.1 was used for plotting. After removing outliers using box plots in SPSS, statistical analyses were performed using nonparametric tests (Mann–Whitney), and Spearman’s test was used in correlation tests. *p* < 0.05 was considered statistically significant.

## 3 Result

### 3.1 Effect of different periods of CUMS on behavioral tests in female C57BL/6N mice

**Figure 3:**
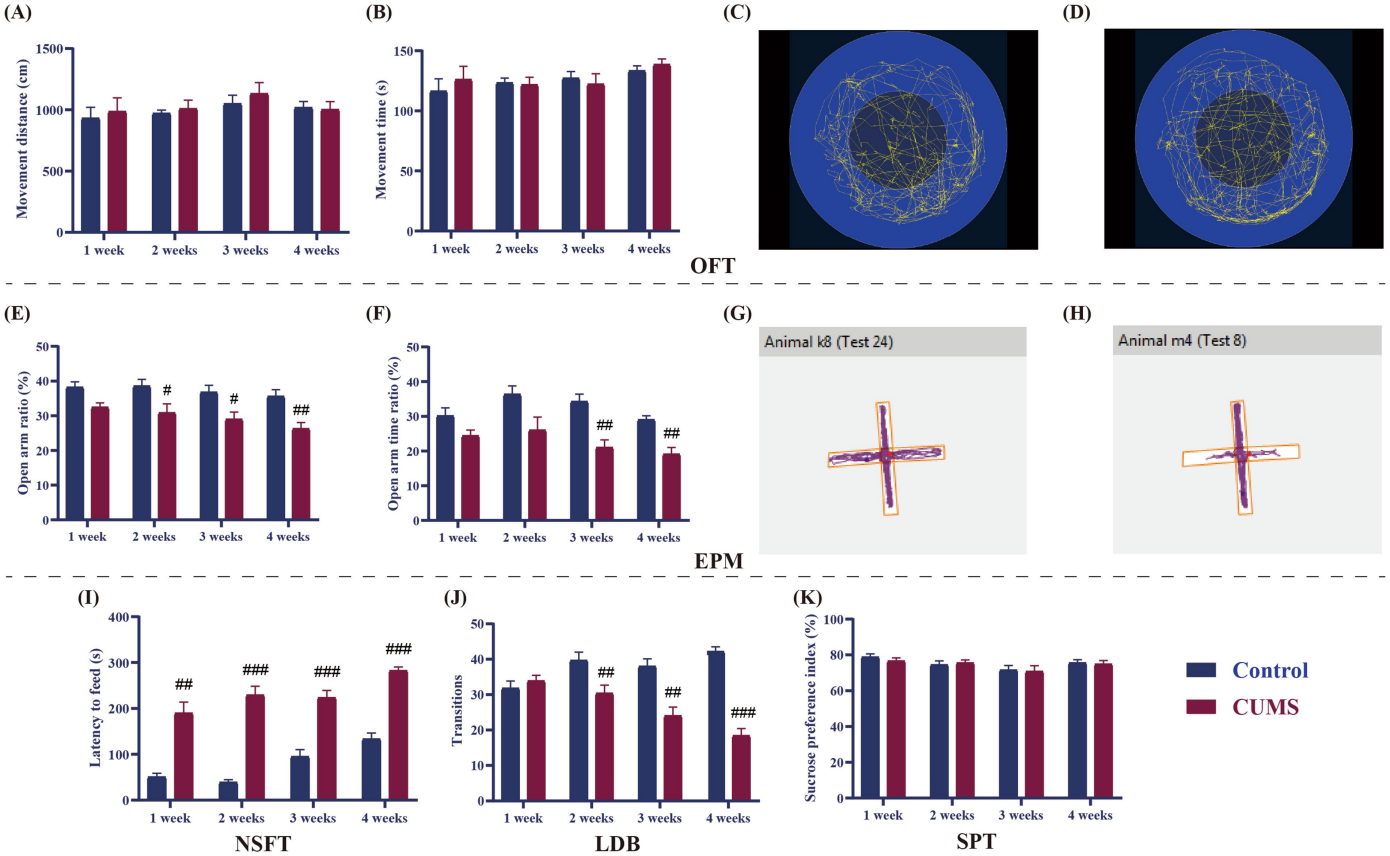
Effect of different periods of CUMS on behavioral tests in female C57BL/6N mice. **(A)**, movement distance; **(B)**, movement time; **(C)**, movement trajectories of control mice in OFT; **(D)**, Movement trajectories of CUMS mice in OFT; **(E)**, open arms ratio (Number of entries into the open arms/ Total number of entries into open and closed arms); **(F)**, open arms time ratio (The time spent with open arms/Total test duration); **(G)**, Movement trajectories of control mice in EPM; **(H)**, Movement trajectories of CUMS mice in EPM; **(I)**, latency to feed; **(J)**, transition; **(K)**, sucrose preference index. N = 8–10, means ± SEM. Compared with control group, ^#^*p* < 0.05, ^##^*p* < 0.01, ^###^*p* < 0.001.

As shown in the results of OFT, there was no difference in movement distance and time between the control and CUMS mice after 1–4 weeks of CUMS (Figures 3A–D).

As shown in Figures 3E,F, following 1 week of CUMS, the open arms ratio and time ratio of CUMS mice in the EPM did not change significantly from those of the control mice. The open arms ratio was much lower than that of the control mice after 2 weeks of CUMS (*p* < 0.05), although the open arms time ratio did not significantly alter in the CUMS mice. The open arms ratio and time ratio of CUMS mice dramatically dropped after 3 and 4 weeks of CUMS in comparison to the control group (*p* < 0.05, *p* < 0.01) (Figures 3G,H).

In the NSFT, compared to the control group, the latency to feed of the CUMS mice was significantly prolonged from CUMS week 1 onwards (*p* < 0.01, *p* < 0.001, Figure 3I).

After 1 week of CUMS, the transition between the CUMS and the control mice did not differ significantly, as shown in the LDB (Figure 3J). The transition in CUMS mice was substantially less than that of the control mice from CUMS week 2 onward (*p* < 0.01, *p* < 0.001, Figure 3F).

Following 1–4 weeks of CUMS, the sucrose preference index of the CUMS mice did not change significantly from that of the control mice in SPT (Figure 3K).

### 3.2 Effect of different periods CUMS on the serum corticosterone (CORT) levels in female C57BL/6N mice

**Figure 4:**
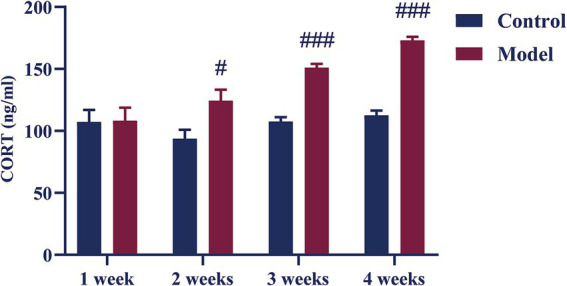
Effect of different periods CUMS on the serum CORT levels in female C57BL/6N mice. *N* = 8–10, means ± SEM. Compared with the control group, ^#^*p* < 0.05, ^###^*p* < 0.001.

As shown in Figure 4, there was no difference in serum CORT content in model mice after 1 week of CUMS compared with the control mice. Compared to the control group the serum CORT content of mice in the model group increased from week 2 onwards (*p* < 0.01, *p* < 0.001).

### 3.3 Effect of different periods CUMS on the relative mRNA expression of pro-inflammatory cytokines in the hippocampus of female C57BL/6N mice

**Figure 5:**
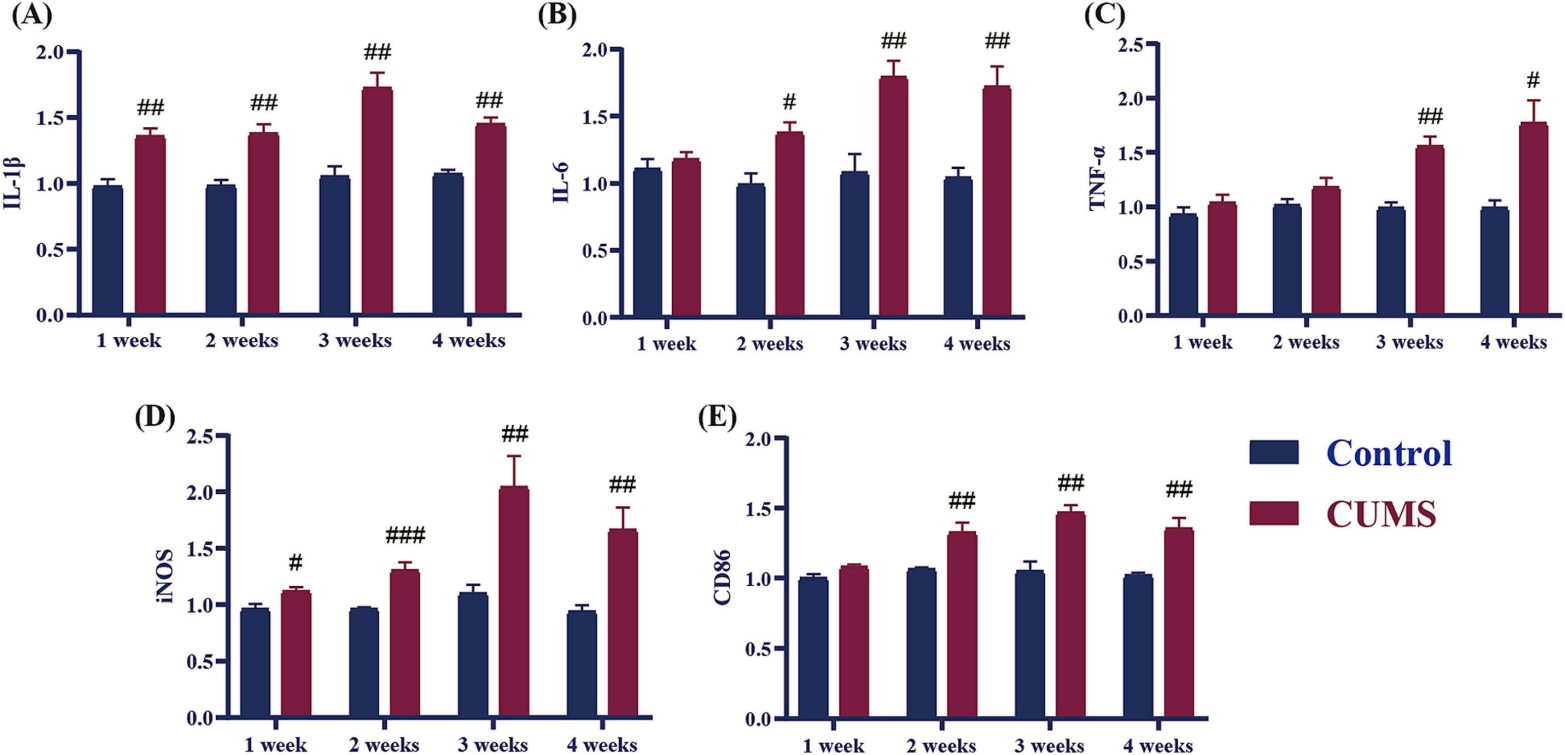
Effect of different periods CUMS on the relative mRNA expression of pro-inflammatory cytokines in the hippocampus of female C57BL/6N mice. **(A)**, IL-1β; **(B)**, IL-6; **(C)**, TNF-α; **(D)**, iNOS; **(E)**, CD86. *N* = 6–8, means ± SEM. Compared with control group, ^#^*p* < 0.05, ^##^*p* < 0.01, ^###^*p* < 0.001.

As it is demonstrated in Figure 5, after 1 week of CUMS, the relative mRNA expression levels of the pro-inflammatory cytokines IL-1β and iNOS of CUMS mice significantly increased in comparison to the control group (*p* < 0.05, *p* < 0.01). In contrast, the relative mRNA expression levels of IL-6, TNF-α, and CD86 did not significantly change in comparison to the control group. After 2 weeks of CUMS, the model group’s relative mRNA expression levels of the pro-inflammatory cytokines IL-1β, IL-6, iNOS, and CD86 were significantly higher than those of the control group (*p* < 0.05, *p* < 0.01, *p* < 0.001), but there was no discernible difference in the relative mRNA expression levels of TNF-α. In comparison to the control group, the model group’s relative mRNA expression levels of IL-1β, IL-6, TNF-α, iNOS, and CD86 increased considerably from CUMS week 3 onwards (*p* < 0.05, *p* < 0.01, *p* < 0.001).

### 3.4 Effect of different periods CUMS on the relative mRNA expression of anti-inflammatory cytokines in the hippocampus of female C57BL/6N mice

**Figure 6:**
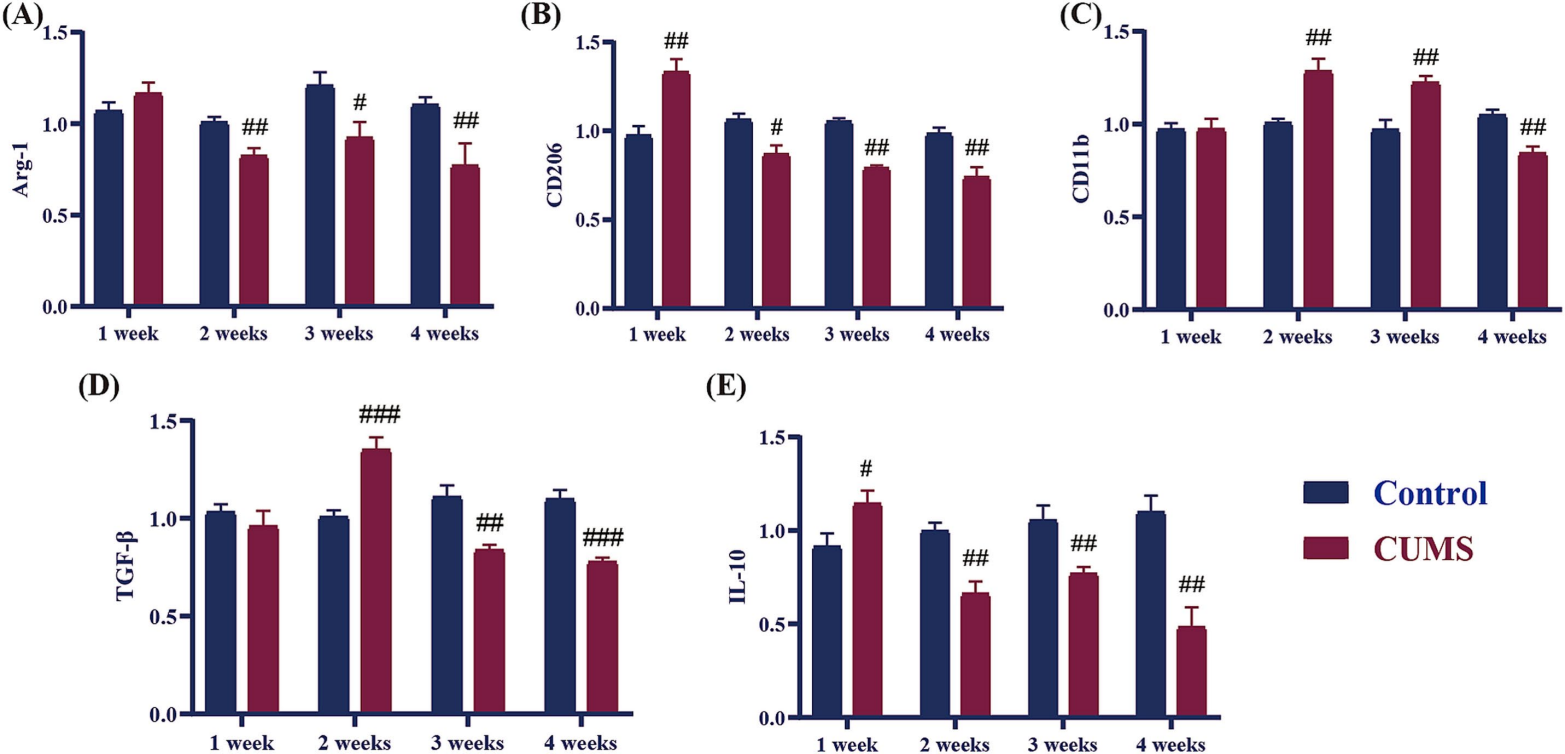
Effect of different periods CUMS on the relative mRNA expression of anti-inflammatory cytokines in the hippocampus of female C57BL/6N mice. **(A)**, Arg-1; **(B)**, CD206; **(C)**, CD11b; **(D)**, TGF-β; **(E)**, IL-10. *N* = 6–8, means ± SEM. Compared with control group, ^#^*p* < 0.05, ^##^*p* < 0.01, ^###^*p* < 0.001.

As shown in Figure 6, following 1 week of CUMS, the mRNA expression levels of the anti-inflammatory cytokines CD206 and IL-10 in CUMS mice significantly increased in comparison to the control group (*p* < 0.05, *p* < 0.01), whereas the Arg-1, TGF-β, and CD11b did not differ significantly from the control group. The anti-inflammatory cytokines TGF-β and CD11b showed significantly higher relative mRNA expression levels in the model group after 2 weeks of CUMS (*p* < 0.01), whereas Arg-1, CD206, and IL-10 showed significantly lower relative mRNA expression levels than in the control group (*p* < 0.05, *p* < 0.01). Following a three-week period of CUMS, the relative mRNA expression levels of the anti-inflammatory cytokine CD11b in the model group exhibited a significant increase compared to the control group (*p* < 0.01). Concurrently, the relative mRNA expression levels of the anti-inflammatory cytokines Arg-1, CD206, TGF-β, and IL-10 mRNA underwent a significant reduction compared to the control group (*p* < 0.05, *p* < 0.01, *p* < 0.001). The relative mRNA expression levels of the anti-inflammatory cytokines Arg-1, CD206, TGF-β, CD11b, and IL-10 in the model group demonstrated a significant decline in comparison to the control group from CUMS week 4 onwards (*p* < 0.01, *p* < 0.001).

### 3.5 Effect of different periods CUMS on tryptophan metabolism in the hippocampus of mice

**Figure 7:**
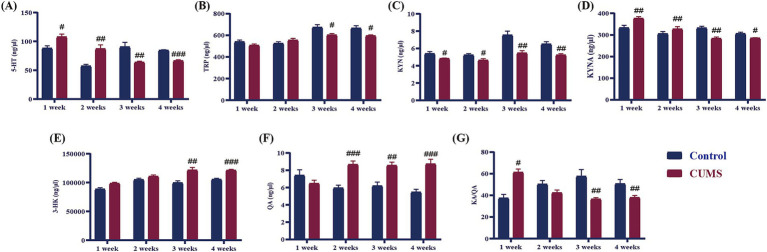
Effect of different periods CUMS on tryptophan metabolism in the hippocampus of mice. **(A)**, 5-HT; **(B)**, TRP; **(C)**, KYN; **(D)**, KYNA; **(E)**, 3-HK; **(F)**, QA; **(G)**, KA/QA. *N* = 8–10, means ± SEM. Compared with control group, ^#^*p* < 0.05, ^##^*p* < 0.01, ^###^*p* < 0.001.

As demonstrated in Figure 7, following 1 week of CUMS, the contents of 5-HT and KYNA, and KA/QA in the hippocampus of the model group exhibited a significant increase in comparison to the control group (*p* < 0.05, *p* < 0.01). Conversely, the levels of TRP, 3-HK, and QA in CUMS mice remained unaltered, and the level of KYN of CUMS mice significantly decreased from CUMS week 1 onwards (*p* < 0.05). After 2 weeks of CUMS, the levels of 5-HT, KYNA, and QA in the model group significantly increased compared to the control group (*p* < 0.05, *p* < 0.01), while the contents of TRP, 3-HK, and KA/QA showed no significant differences compared to the control group. In contrast, the levels of 5-HT, TRP, KYN, KYNA, and KA/QA in the hippocampus of the mice exhibited a significant decline (*p* < 0.05, *p* < 0.01, *p* < 0.001), while the levels of 3-HK and QA demonstrated a significant increase from CUMS week 3 onwards (*p* < 0.01, *p* < 0.001).

### 3.6 Effects of different periods of CUMS on neurotransmitters in the hippocampus

**Figure 8:**
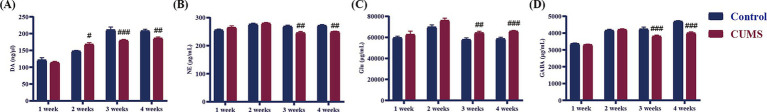
Effects of different periods of CUMS on neurotransmitters in the hippocampus. **(A)**, DA; **(B)**, NE; **(C)**, Glu; **(D)**, GABA. *N* = 8–10, means ± SEM. Compared with control group, ^#^*p* < 0.05, ^##^*p* < 0.01, ^###^*p* < 0.001.

As displayed in Figure 8, after giving CUMS 1 week, there was no significant difference in the levels of DA, NE, Glu, and GABA in the hippocampus of CUMS mice compared to the control mice. After 2 weeks of CUMS, the level of DA in the hippocampus of the CUMS mice significantly rose compared to the control group (*p* < 0.01), while the levels of NE, Glu, and GABA showed no significant difference. After 3–4 weeks of CUMS, the levels of DA, NE, and GABA in the hippocampus of the model group mice significantly decreased compared to the control group (*p* < 0.01, *p* < 0.001), while the level of Glu significantly enhanced (*p* < 0.01).

### 3.7 Correlation analysis of anxiety-like behavior indicators, inflammatory cytokines, tryptophan metabolites, and neurotransmitter levels in female C57BL/6N mice subjected to CUMS for 4 weeks

**Figure 9:**
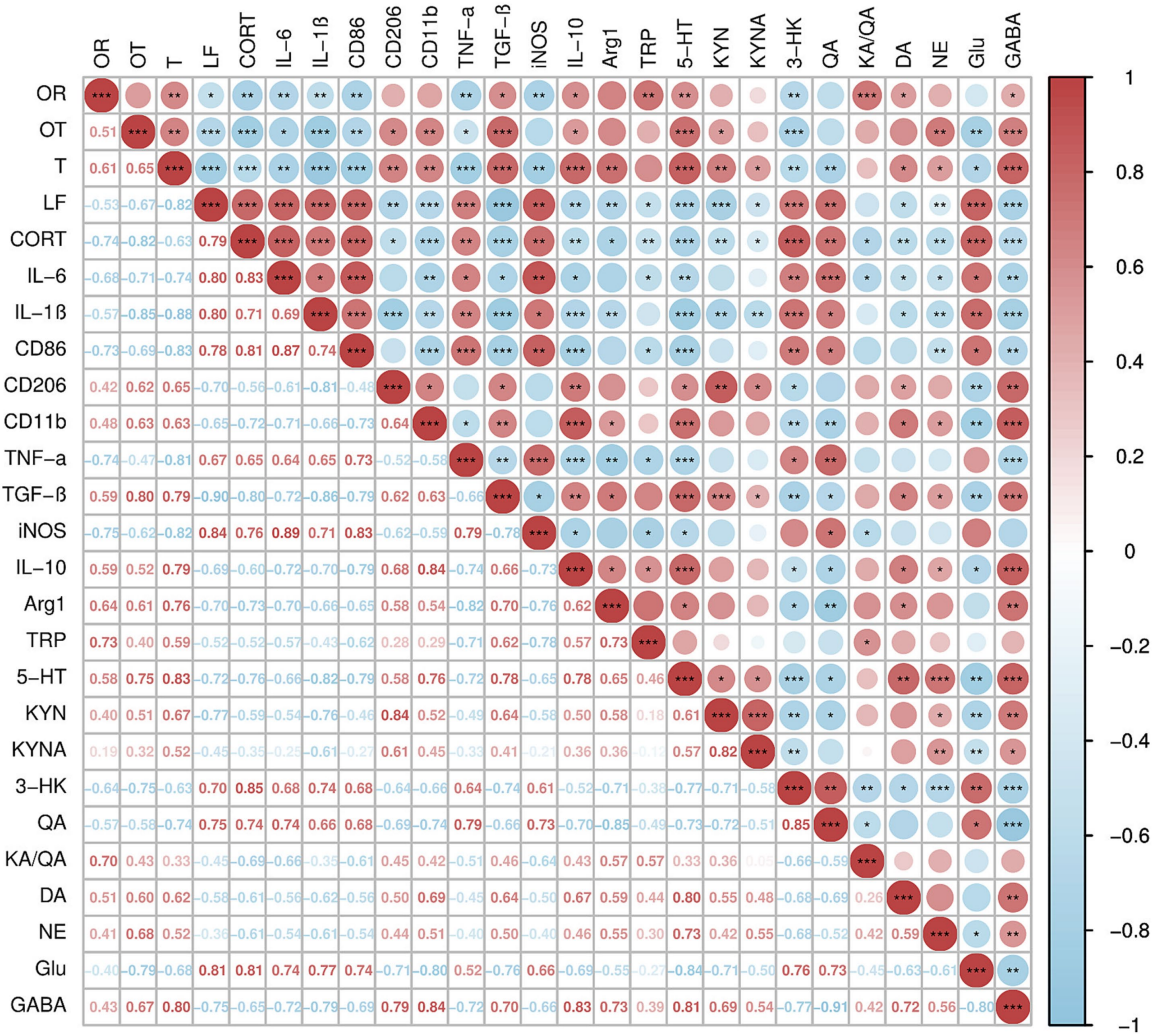
Correlation analysis of anxiety-like behavior indicators, inflammatory cytokines, tryptophan metabolites, and neurotransmitter levels in female C57BL/6N mice subjected to CUMS for 4 weeks. *N* = 16, means ± SEM, *p < 0.05, **p < 0.01, ***p < 0.001. Anxiety-like behavior indicators: OR, open arms ratio; OT, open arms time ratio; T, transition; LF, latency to feed. Inflammatory cytokines: IL-6, IL-1β, CD86, CD206, CD11b, TNF-α, TNF-β, iNOS, IL-10, Arg-1. Tryptophan metabolites: TRP, 5-HT, KYN, KYNA, 3-HK, QA, KA/QA. Neurotransmitter levels: DA, NE, Glu, GABA. Red represents positive correlation, blue represents negative correlation, the higher the correlation, the deeper the color, and vice versa.

A correlation analysis was conducted using Spearman’s correlation coefficient on the anxiety-like behavioral indicators, inflammatory cytokines, tryptophan metabolites, and neurotransmitter levels of mice subjected to CUMS stress for 4 weeks. As shown in Figure 9, the open arms ratio (OR) in the EPM showed a positive correlation with inflammatory cytokines (TGF-β, IL-10), tryptophan metabolites (TRP, 5-HT, KA/QA), and neurotransmitters (DA, GABA) (*r* = 0.59, *p* < 0.05; *r* = 0.59, *p* < 0.05; *r* = 0.73, *p* < 0.01; *r* = 0.58, *p* < 0.01; *r* = 0.70, *p* < 0.001; *r* = 0.51, *p* < 0.05; *r* = 0.43, *p* < 0.05); and a negative correlation with CORT levels, inflammatory cytokines (IL-6, IL-1β, CD86, TNF-α, iNOS), and the tryptophan metabolite 3-HK (*r* = −0.74, *p* < 0.01; *r* = −0.68, *p* < 0.01; *r* = −0.57, *p* < 0.01; *r* = −0.73, *p* < 0.01; *r* = −0.74, *p* < 0.01; *r* = −0.75, *p* < 0.01; *r* = −0.64, *p* < 0.01). The open arms time ratio (OT) showed a positive correlation with inflammatory cytokines (CD206, CD11b, TGF-β, IL-10), tryptophan metabolites (5-HT, KYN), and neurotransmitters (NE, GABA) (*r* = 0.62, *p* < 0.05; *r* = 0.63, *p* < 0.01; *r* = 0.80, *p* < 0.001; *r* = 0.52, *p* < 0.05; *r* = 0.75, *p* < 0.001; *r* = 0.51, *p* < 0.05; *r* = 0.68, *p* < 0.01; *r* = 0.67, *p* < 0.001); and a negative correlation with CORT levels, inflammatory cytokines (IL-6, IL-1β, CD86, TNF-α), the tryptophan metabolite 3-HK, and the neurotransmitter Glu (*r* = −0.82, *p* < 0.001; *r* = −0.71, *p* < 0.05; *r* = −0.85, *p* < 0.001; *r* = −0.69, *p* < 0.01; *r* = −0.47, *p* < 0.05; *r* = −0.75, *p* < 0.001; *r* = −0.69, *p* < 0.01). The transition (T) in the LDB showed a positive correlation with inflammatory cytokines (CD206, CD11b, TGF-β, IL-10, Arg-1), tryptophan metabolites (5-HT, KYN, KYNA), and neurotransmitters (DA, NE, GABA) (*r* = 0.65, *p* < 0.01; *r* = 0.63, *p* < 0.01; *r* = 0.79, *p* < 0.001; *r* = 0.79, *p* < 0.001; *r* = 0.76, *p* < 0.01; *r* = 0.83, *p* < 0.001; *r* = 0.67, *p* < 0.01; *r* = 0.52, *p* < 0.05; *r* = 0.62, *p* < 0.05; *r* = 0.52, *p* < 0.05; *r* = 0.80, *p* < 0.001); negatively correlated with CORT content, inflammatory cytokines (IL-6, IL-1β, CD86, TNF-α, iNOS), tryptophan metabolites 3-HK, QA, and neurotransmitter Glu (*r* = −0.63, *p* < 0.001; *r* = −0.74, *p* < 0.01; *r* = −0.88, *p* < 0.001; *r* = −0.83, *p* < 0.001; *r* = −0.81, *p* < 0.001; *r* = −0.82, *p* < 0.01; *r* = −0.63, *p* < 0.01; *r* = −0.74, *p* < 0.01; *r* = −0.68, *p* < 0.05). The latency to feed (LF) in NSFT showed a positive correlation with CORT content, inflammatory cytokines (IL-6, IL-1β, CD86, TNF-α, iNOS), tryptophan metabolites 3-HK, QA, and neurotransmitter Glu (*r* = 0.79, *p* < 0.001; *r* = 0.80, *p* < 0.001; *r* = 0.80, *p* < 0.001; *r* = 0.78, *p* < 0.001; *r* = 0.67, *p* < 0.001; *r* = 0.84, *p* < 0.01; *r* = 0.70, *p* < 0.001; *r* = 0.75, *p* < 0.01; *r* = 0.81, *p* < 0.001); negatively correlated with inflammatory cytokines (CD206, CD11b, TGF-β, IL-10, Arg-1), tryptophan metabolites TRP, 5-HT, KYN, KYNA, and neurotransmitters (DA, NE, GABA) (*r* = −0.75, *p* < 0.01; *r* = −0.60, *p* < 0.001; *r* = −0.90, *p* < 0.001; *r* = −0.69, *p* < 0.01; *r* = −0.70, *p* < 0.01; *r* = −0.52, *p* < 0.05; *r* = −0.72, *p* < 0.001; *r* = −0.77, *p* < 0.001; *r* = −0.45, *p* < 0.05; *r* = −0.58, *p* < 0.05; *r* = −0.36, *p* < 0.01; *r* = −0.75, *p* < 0.001).

## 4 Discussion

The CUMS model is widely used to induce anxiety or depressive-like behavior in animals through prolonged exposure to unpredictable stressors, which stimulate the various stressors humans encounter in daily life (Antoniuk et al., 2019). This model effectively triggers anxiety-like symptoms in animals, mirroring the experiences of humans with anxiety disorders. In our investigation, female C57BL/6N mice were subjected to CUMS to assess the stress duration required for the emergence of anxiety-like behaviors. Consistent with earlier research, our results for CUMS-exposed mice and control animals did not significantly differ in movement distance or time spent moving, suggesting that CUMS did not affect voluntary motor performance (Wang et al., 2021). In current study, after 2 weeks of CUMS, female C57BL/6N mice showed reduced the open arms ratio in EPM, prolonged the latency to feed in NSFT, and decreased the transition in LDB. The above experimental results indicate that female mice would exhibit anxiety-like behaviors after 2-week stress, which would be more severe after 3 and 4 weeks of stress exposure. However, there was no significant difference in sucrose preference index between the CUMS and control groups throughout the 1- to 4-week period, indicating that depressive-like behaviors did not emerge in female C57BL/6N mice during this period. In line with previous research (Yan et al., 2021), female mice often develop depressive-like behavior after 5 weeks of CUMS administration. In summary, the behavioral findings of this study indicate that 2 to 4 weeks of CUMS exposure induces anxiety-like behaviors in female C57BL/6N mice, whereas depressive-like behaviors appear only after 5 weeks of stress. These findings shed light on the temporal development of anxiety and depressive-like symptoms in this model, supporting its application in the anxiety related disorders.

Long-term chronic stress has been demonstrated to cause hyperactivation of the hypothalamic–pituitary–adrenal (HPA) axis, resulting in increased glucocorticoid release, which can alter mood and behavior. Anxiety disorders are frequently associated with elevated levels of serum CORT (Hasler et al., 2004; Gądek-Michalska et al., 2013). In the present study, female C57BL/6N mice showed an anxiety-like behavior accompanying with a significantly higher blood CORT levels after 2–4 weeks of CUMS, indicating a hyperactive HPA axis in these animals at the time. This stimulation of the HPA axis affects the homeostasis of neural circuits and can interfere with the function of numerous essential monoamine neurotransmitters, including norepinephrine, serotonin (5-HT), and dopamine (Xu et al., 2017). Additionally, ovariectomized female mice with reduced DA, 5-HT, and NE levels exhibit anxiety-like symptoms (Izumo et al., 2012). In this study, we evaluated the levels of 5-HT, DA, and NE in the hippocampus, and there was a significant decrease in these neurotransmitters after 3 weeks of CUMS, indicating that HPA axis hyperactivity affects monoamine neurotransmitter function in female mice. At the same time, the decrease in the levels of 5-HT, DA, and NE in the hippocampus is an important feature of anxiety disorders (Vilela et al., 2021). In addition to monoamines, Glu and GABA play crucial roles in the pathogenesis of anxiety disorders (Ende, 2015). Glu is an excitatory neurotransmitter whose levels in the brain have been associated with the severity of anxiety symptoms (Brehl et al., 2020). Excessive Glu formation in the central nervous system can cause neuronal injury, and higher Glu levels have been found in patients with anxiety disorders, disrupting the balance of excitatory and inhibitory neurotransmission (Mehta et al., 2013; Zacharopoulos et al., 2022). On the other hand, GABA is a primary inhibitory neurotransmitter, and its role in mitigating anxiety is well established by its ability to counteract the excessive neuronal excitation associated with emotional regulation (Lydiard, 2003; Nemeroff, 2003). In this study, after 3 weeks of CUMS, the hippocampal levels of Glu were significantly elevated, while GABA levels were reduced, indicating a disrupted balance between these two neurotransmitters. This disruption suggests that chronic stress induces an imbalance between excitatory and inhibitory neurotransmission, potentially contributing to the anxiety-like behaviors observed in the female C57BL/6N mice.

Chronic long-term stress has been shown to elevate inflammation levels both in the brain and periphery, contributing to the development of various neuropsychiatric disorders (Glaser and Kiecolt-Glaser, 2005). Cytokines, which are essential signaling molecules in immune responses and inflammation, play a pivotal role in these processes (Bhol et al., 2024). Clinically, patients with anxiety disorders often exhibit significantly elevated levels of pro-inflammatory cytokines (Gualtieri et al., 2020; Vijay et al., 2024). The delicate balance between pro-inflammatory and anti-inflammatory cytokines is crucial for maintaining immune system function and facilitating tissue repair (Bhol et al., 2024). Disruption of this balance by excessive pro-inflammatory cytokine expression and relative deficiency in anti-inflammatory cytokines can result in chronic inflammation, which is thought to trigger or exacerbate anxiety disorders (Hou et al., 2017). In our study, we observed changes in cytokine levels throughout CUMS exposure in female mice. At the early stage (1 week), some pro-inflammatory cytokines, such as IL-1β and iNOS, and some anti-inflammatory cytokines, including CD206 and IL-10, began to rise. After 2 weeks of CUMS, we found an increase in pro-inflammatory cytokines (IL-6, CD86) and some anti-inflammatory cytokines (CD11b, TGF-β), while the levels of certain anti-inflammatory cytokines, such as Arg-1, CD206, and IL-10, began to decrease. By 3 weeks of CUMS, the levels of anti-inflammatory cytokines (TGF-β, Arg-1, CD206, IL-10) continued to decrease, while pro-inflammatory cytokines (IL-1β, IL-6, iNOS, CD86, TNF-α) showed a persistent rise. At 4 weeks, this trend continued, with pro-inflammatory cytokines remaining elevated and anti-inflammatory cytokines still reduced. These findings indicate that during the early stages of CUMS (1–2 weeks), both pro-inflammatory and anti-inflammatory cytokines are released simultaneously in the hippocampus of female C57BL/6N mice. However, as stress persists beyond 3 weeks, the secretion of anti-inflammatory cytokines decreases, while pro-inflammatory cytokine levels continue to increase. Studies have shown that chronic stress in female C57BL/6N mice leads to elevated levels of pro-inflammatory cytokines, such as IL-1β, IL-6, and TNF-α, which is consistent with our findings (Helman et al., 2022). This shift disrupts the relative balance between pro-inflammatory and anti-inflammatory cytokines, exacerbating the inflammatory response. Furthermore, behavioral data from earlier stages of the study suggest that after 3 weeks of CUMS, female C57BL/6N mice begin to exhibit anxiety-like behaviors. This behavioral shift correlates with the imbalance in cytokine levels, indicating a potential link between chronic stress-induced inflammation and the onset of anxiety in this model.

In the inflammatory response, pro-inflammatory cytokines activate the enzyme indoleamine 2,3-dioxygenase (IDO), which plays a crucial role in the kynurenine pathway, promoting the metabolism of tryptophan (Schwarcz et al., 2012). In current study, after 1 week of CUMS in female mice, inflammatory responses began to occur in the hippocampus, and KYN levels started to decline, indicating that the kynurenine pathway was activated. The kynurenine pathway is further divided into two main branches: KYN-KYNA and KYN-QA (Kanai et al., 2009). The KYN-KYNA branch converts KYN to KYNA, a neuroprotective metabolite, while the KYN-QA branch converts KYN into 3-HK, which is further metabolized into QA (Foster et al., 1985; Han et al., 2010; Schwarcz et al., 2012). In our study, we observed that during the early stage of CUMS (1 week), the kynurenine pathway in female C57BL/6N mice favored the KYN-KYNA branch, leading to an increase in KYNA production, while the neurotoxic metabolites 3-HK and QA did not show significant accumulation. By the second week of CUMS, KYNA levels continued to rise, and at this point, QA began to accumulate, indicating an early shift towards the KYN-QA branch. After 3–4 weeks of CUMS exposure, this shift became more pronounced, with a marked decrease in KYNA levels and a significant increase in the production of the neurotoxic substances 3-HK and QA. Under normal conditions, these two branches of the kynurenine pathway function independently and maintain a balanced equilibrium. However, this balance is disrupted during inflammatory responses. The body requires significant amounts of energy to combat inflammation, which leads to a shift in the kynurenine pathway towards the KYN-QA branch. Previous studies have also shown that after 4 weeks of stress in mice, tryptophan metabolism in the hippocampus shifted more towards KYN-QA, with an accumulation of 3-HK and QA content, which is consistent with our research findings (Ye et al., 2024). The above results suggest that tryptophan metabolism was progressively disrupted, leading to an imbalance in the kynurenine pathway and an accumulation of neurotoxic metabolites. This disruption may contribute to the neuroinflammatory response and the onset of anxiety-like behaviors observed in the mice.

In clinical studies, patients with anxiety disorder often exhibit reduced 5-HT and GABA levels, alongside increased CORT and inflammatory factors (IL-6, TNF-α) (Paris, 2018; Vismara et al., 2020; Łoś and Waszkiewicz, 2021). In this study, we conducted a correlation analysis of the anxiety behavior indicators of female mice that underwent 4 weeks of CUMS with CORT levels, tryptophan metabolites, neurotransmitter content, and the relative mRNA expression of inflammatory factors. The results show a significant correlation between anxiety behavioral indicators and CORT levels, inflammatory factors (IL-6, IL-1β, CD86, TNF-α, TGF-β, IL-10), 5-HT, and GABA. This suggests that CORT, inflammatory factors (IL-6, IL-1β, CD86, TNF-α, TGF-β, IL-10), 5-HT, and GABA may serve as biomarkers for anxiety disorders.

## 5 Conclusion

Different periods of CUMS significantly impact anxiety-like behavior, inflammatory response, and the kynurenine pathway in tryptophan metabolism in female C57BL/6N mice. These results demonstrate that female mice did not exhibit anxiety-like behavior after 1 week of CUMS, and the inflammatory responses commenced early in the onset of anxiety-like behavior in female mice, while the two branches of the kynurenine pathway remained relatively balanced. As anxiety-like behavior appeared and persisted, inflammation in the hippocampus of female mice intensified, leading to a disruption in tryptophan metabolism. This progressive shift suggests that prolonged exposure to stress may not only enhance neuroinflammation but also alter metabolic processes, contributing to the development and persistence of anxiety-like behaviors.

## Data Availability

The original contributions presented in the study are included in the article/supplementary material, further inquiries can be directed to the corresponding authors.

## Glossary

CUMS: chronic unpredictable mild stress
OFT: open field test
EMP: elevated plus maze
NSFT: novelty-suppressed feeding test
LDB: light/dark box
SPT: sucrose preference test
IL-6: interleukin 6
IL-1β: interleukin-1β
iNOS: inducible nitric oxide synthase
CD86: cluster of differentiation 86
TNF-α: tumor necrosis factor-α
TGF-β: transforming growth factor-β
CD206: Macrophage mannose receptor 1
CD11b: CD11 antigen-like family member B
Arg-1: arginase-1
5-HT: 5-hydroxy tryptamine
TRP: tryptophan
KYN: kynurenine
KYNA: kynurenic acid
3-HK: 3-hydroxykynurenine
QA: quinolinic acid
DA: dopamine
NE: norepinephrine
Glu: glutamate
GABA: *γ*-aminobutyric acid
